# Long working hours and metabolic syndrome among Japanese men: a cross-sectional study

**DOI:** 10.1186/1471-2458-12-395

**Published:** 2012-05-31

**Authors:** Tomoko Kobayashi, Etsuji Suzuki, Soshi Takao, Hiroyuki Doi

**Affiliations:** 1 Department of Epidemiology, Okayama University Graduate School of Medicine, Dentistry and Pharmaceutical Sciences, 2-5-1 Shikata-cho, Kita-ku, Okayama, 700-8558, Japan

**Keywords:** Long working hours, Metabolic syndrome, Japan, Trigger level

## Abstract

**Background:**

The link between long working hours and health has been extensively studied for decades. Despite global concern regarding metabolic syndrome, however, no studies to date have solely evaluated the relationship between long working hours and that syndrome. We therefore examined the association between long working hours and metabolic syndrome in a cross-sectional study.

**Methods:**

Between May and October 2009, we collected data from annual health checkups and questionnaires from employees at a manufacturing company in Shizuoka, Japan. Questionnaires were returned by 1,601 workers (response rate: 96.2%; 1,314 men, 287 women). After exclusions, including women because of a lack of overtime work, the analysis was performed for 933 men. We calculated odds ratios (ORs) and 95% confidence intervals (CIs) for metabolic syndrome. Further, we conducted a stratified analysis by age-group (<40 years vs. ≥40 years).

**Results:**

Metabolic syndrome was identified in 110 workers (11.8%). We observed a positive association between working hours and metabolic syndrome after adjusting for age, occupation, shift work, smoking status, frequency of alcohol consumption, and cohabiting status. Compared with subjects who worked 7–8 h/day, multivariate ORs for metabolic syndrome were 1.66 (95% CI, 0.91–3.01), 1.48 (95% CI, 0.75–2.90), and 2.32 (95% CI, 1.04–5.16) for those working 8–9 h/day, 9–10 h/day, and >10 h/day, respectively. Similar patterns were obtained when we excluded shift workers from the analysis. In age-stratified analysis, the corresponding ORs among workers aged ≥40 years were 2.02 (95% CI, 1.04–3.90), 1.21 (95% CI, 0.53–2.77), and 3.14 (95% CI, 1.24–7.95). In contrast, no clear association was found among workers aged <40 years.

**Conclusions:**

The present study suggests that 10 h/day may be a trigger level of working hours for increased risk of metabolic syndrome among Japanese male workers.

## Background

Metabolic syndrome is one of the most serious public health concerns globally, including developing countries [1]. Recent research has indicated that metabolic syndrome is highly predictive of cardiovascular disease, myocardial infarction, stroke, and all-cause mortality [2]. Although obesity used to be relatively rare in the Japanese population, the number of overweight male adults has increased over the last few decades [3]. Indeed, the prevalence of metabolic syndrome increases dramatically with age—from 8.5% among subjects in their 30s to over 35% in subjects older than 60 years [4]—and metabolic syndrome is becoming a major public health issue in Japan.

It is now well established that the work environment exerts both beneficial and detrimental health effects among the working population [5]. In fact, long working hours have been considered one of the critical determinants of health in occupational medicine, and *karoshi* (sudden death due to cardiovascular events resulting from working overtime) was first recognized in Japan [6]. Previous studies have examined the effects of long working hours on various health outcomes, such as body mass index and waist circumference [7,8], obesity [9], hypertension [10-12], diabetes [13-15], acute myocardial infarction [16], and coronary heart disease [17-19]. Although most studies indicated an adverse effect of long working hours on health, the evidence remains sparse, and some studies even reported beneficial health effects of long working hours [8,11,15]. A previous review thus concluded that the evidence is still inconclusive and that more epidemiological studies are needed to clarify the different effects of long working hours [20]. Despite global concern regarding metabolic syndrome, however, no studies to date have solely evaluated the relationship between long working hours and metabolic syndrome. Although one study reported that overtime combined with midnight shift work may be a risk factor for metabolic syndrome, that study was based only on 98 police officers in the United States [21]. Since metabolic syndrome comprises a cluster of risk factors for cardiovascular disease and type 2 diabetes mellitus, including abnormal obesity and insulin resistance [22], long working hours may increase the risk of metabolic syndrome through several pathways (e.g., changes in physiological processes and health-related behavior).

The aim of the present study was to investigate the association between long working hours and metabolic syndrome among Japanese workers.

## Methods

### Participants

The cross-sectional study was performed among regular Japanese workers aged 19 to 70 years at a manufacturing company in Shizuoka Prefecture. In late May 2009, we collected data from questionnaires, which included items on sociodemographic factors, working conditions, and health-related behavior. We also collected data relating to health outcome from the workers’ annual health checkup, which was conducted as a legal requirement between May and October 2009. Almost all the subjects responded to the questionnaires before the health checkup: of the 1,664 study subjects, 1,601 returned the questionnaire, and the response rate was 96.2% (1,314 men, 287 women). We restricted the analysis to men since 78.8% of the women did not work overtime, which precluded further analysis between working hours and metabolic syndrome among women. Of the remaining 1,314 men, we excluded participants who did not respond to the questionnaire item on working hours (*n* = 27), those who worked fewer than 7 h/day (*n* = 2), and those who for administrative reasons lacked annual health checkup information, e.g., unavailability of electronic data relating to the checkup (*n* = 352). Thus, 933 men were included in the analysis.

The study was reviewed and approved by the Ethics Committee on the Research of Epidemiology at Okayama University Graduate School of Medicine, Dentistry and Pharmaceutical Sciences.

### Measures

Average working hours were ascertained using the following question: “How many hours per day did you work over the past month on average?” We divided the participants into the following four categories of working hours: ≥7 to 8 h/day, >8 to 9 h/day, >9 to 10 h/day, and >10 h/day. We also determined whether the subjects undertook shift work or not.

We applied the Japanese criteria for metabolic syndrome [23] except with respect to medication, details of which were not available in the present study. Thus, metabolic syndrome was defined as having a waist circumference of ≥85 cm and at least two of the following three conditions: (1) high serum triglyceride (≥150 mg/dl) and/or low high-density lipoprotein (HDL) cholesterol (<40 mg/l); (2) high blood pressure (≥130/85 mmHg); and (3) high fasting plasma glucose (≥110 mg/dl). Waist circumference was measured at the umbilical level using non-elastic tape by a medical staff member. Serum triglyceride, HDL, and fasting plasma glucose were measured with fasting blood specimens using, respectively, a glycerol removal method, an enzymatic method, and a stepwise high-performance liquid chromatography method. Blood pressure was measured with the subject in a sitting position by means of a sphygmomanometer on the arm. Each subject was required to undergo a health checkup in the morning of a day specified by the company; to obtain accurate measures, they were also required to observe an overnight fast (no food after 9 p.m.).

Sociodemographic factors included age, cohabiting status (cohabiting vs. non-cohabiting), and occupation (clerical, technical, skilled, sales, and others). Health-related behavior included smoking status (current vs. never/former), frequency of alcohol consumption, and average sleeping hours. Frequency of alcohol consumption was categorized into the following three groups: none/rarely, sometimes (1–3 days/month, 1–3 days/week), and often (4–6 days/week, every day). Average sleeping hours were obtained using the following question: “How many hours did you sleep per day over the past month on average?”

### Statistical analyses

We first examined the relationship between working hours and metabolic syndrome by crude logistic regression analysis (Model 1). Then, in Model 2, we adjusted for age (continuous). In Model 3, we additionally adjusted for the following variables with reference to previous studies: occupation [24], shift work [25], smoking status [26,27], frequency of alcohol consumption [28], and cohabiting status [29]. Categorical variables were included as dummy variables, and subjects with missing values were excluded from the analysis. We did not adjust for sleeping hours in Model 3 since sleeping hours were treated as a mediator of the association between long working hours and metabolic syndrome [30]. As a supplementary analysis, we restricted the subjects to non-shift workers to examine whether the associations between long working hours and metabolic syndrome were modified by shift work.

Furthermore, we conducted a stratified analysis by age (<40 years vs. ≥40 years) following the Act on Assurance of Medical Care for Elderly People in Japan [23], which focused on metabolic syndrome among individuals aged 40–74 years.

Finally, we restricted the analyses a posteriori to technicians or skilled workers to examine the possible effect of modification across occupations.

Odds ratios (ORs) and 95% confidence intervals (CIs) for metabolic syndrome were calculated, and a CI excluding 1 was considered statistically significant. All analyses were performed using STATA 12.1 (StataCorp, College Station, TX, USA).

## Results

The sociodemographic and health characteristics of the participants according to their working hours are shown in Table 1. A total of 110 workers (11.8%) met the criteria for metabolic syndrome. Compared with subjects doing no overtime work (7–8 h/day), those with long working hours were more likely to be technicians, non-shift workers, nonsmokers, and short sleepers.

**Table 1:** Characteristics of the study subjects (all males) by working hours, Japan, 2009

	**7–8 hours/day**		**8–9 hours/day**		**9–10 hours/day**		**>10 hours/day**	
	**(*n* = 392)**		**(*n* = 177)**		**(*n* = 196)**		**(*n* = 168)**	
**Characteristics**	**No.**	**%**	**No.**	**%**	**No.**	**%**	**No.**	**%**
Age (years; mean, SD)	44.7	11.1	45.4	10.7	42.6	10.3	40.9	8.9
Occupation								
Clerical	14	3.6	38	21.5	27	13.8	14	8.3
Technical	33	8.4	60	33.9	113	57.7	141	83.9
Skilled	336	85.7	73	41.2	54	27.6	0	0.0
Sales	5	1.3	4	2.3	2	1.0	2	1.2
Others	3	0.8	2	1.1	0	0.0	11	6.6
Missing	1	0.3	0	0.0	0	0.0	0	0.0
Shift work								
Non-Shift worker	253	64.5	128	72.3	148	75.5	159	94.6
Shift worker	138	35.2	48	27.1	47	24.0	8	4.8
Missing	1	0.3	1	0.6	1	0.5	1	0.6
Smoking status								
Never/Former	170	43.4	87	49.2	114	58.2	110	65.5
Current	222	56.6	90	50.9	82	41.8	58	34.5
Frequency of alcohol consumption ^a^								
None/Rarely	129	32.9	49	27.7	40	20.4	45	26.8
Sometimes	90	23.0	55	31.1	64	32.7	74	44.1
Often	173	44.1	73	41.2	91	46.4	49	29.2
Missing	0	0.0	0	0.0	1	0.5	0	0.0
Cohabiting status								
Cohabiting	302	77.0	147	83.1	153	78.1	129	76.8
Non-cohabiting	77	19.6	29	16.4	41	20.9	39	23.2
Missing	13	3.3	1	0.6	2	1.0	0	0.0
Sleeping (hours/day; mean, SD)	6.6	1.0	6.5	0.9	6.2	0.8	5.6	0.8
Metabolic syndrome								
No	352	89.8	150	84.8	173	88.3	148	88.1
Yes	40	10.2	27	15.3	23	11.7	20	11.9

*SD* standard deviation.

^a^ Frequency of alcohol consumption categories are as follows: none/rarely, sometimes (1–3 days/month, 1–3 days/week), and often (4–6 days/week, everyday).

Table 2 shows ORs and 95% CIs for metabolic syndrome. In Models 1 and 2, no statistically significant associations were observed, although the point estimates of ORs among workers with longer working hours were all above 1. When we adjusted for health-related behavior (i.e., smoking status and frequency of alcohol consumption) in addition to age, the ORs did not change substantially. When we subsequently adjusted for shift work and cohabiting status, the ORs of the 8–9 working hours/day and >10 h/day slightly increased. Finally, when we additionally adjusted for occupation, the positive association between working hours and metabolic syndrome was enhanced (Model 3); those who worked >10 h/day had more than double the odds of metabolic syndrome (OR, 2.32; 95% CI, 1.04–5.16). When we additionally adjusted for sleeping hours (continuous) as a supplementary analysis, the positive association was slightly attenuated: the ORs for 8–9, 9–10, and >10 working hours/day were 1.61 (95% CI, 0.88–2.92), 1.36 (95% CI, 0.69–2.68), and 1.86 (95% CI, 0.82–4.22), respectively. When we excluded shift workers from the analysis, the results were similar to those in Table 2: compared with subjects who worked 7–8 h/day, the OR among those who worked >10 h/day was 2.28 (95% CI, 0.88–5.90) (Additional file 1: Table S1).

**Table 2:** Odds ratios for metabolic syndrome associated with working hours among men in Japan, 2009

	**Model 1 ^a^**		**Model 2 ^b^**		**Model 3 ^c^**	
	**(*n* = 933)**		**(*n* = 933)**		**(*n* = 911)**	
**Working hours**	**OR**	**(95% CI)**	**OR**	**(95% CI)**	**OR**	**(95% CI)**
≥7 to 8 hours/day	1.00	Reference	1.00	Reference	1.00	Reference
>8 to 9 hours/day	1.58	(0.94–2.68)	1.56	(0.92–2.66)	1.66	(0.91–3.01)
>9 to 10 hours/day	1.17	(0.68–2.02)	1.31	(0.75–2.28)	1.48	(0.75–2.90)
>10 hours/day	1.19	(0.67–2.10)	1.47	(0.82–2.63)	2.32	(1.04–5.16)

*CI* confidence interval, *OR* odds ratio.

^a^ Crude model.

^b^ Adjusted for age (continuous).

^c^ Adjusted for age (continuous), occupation, shift work, smoking status, frequency of alcohol consumption, and cohabiting status.

When we further stratified the subjects by age (Table 3), we observed a significant association between working hours and metabolic syndrome only among older subjects: in workers aged ≥40 years, the multivariate ORs among those who worked 8–9 h/day and those working >10 h/day were 2.02 (95% CI, 1.04–3.90) and 3.14 (95% CI, 1.24–7.95), respectively, compared with those who worked 7–8 h/day. In contrast, no clear association was found among younger workers <40 years. Similar patterns were observed when we stratified the subjects using other cut-off values (e.g., 45 years and 50 years, as shown, respectively, in Additional file 1: Tables S2 and S3).

**Table 3:** Odds ratios for metabolic syndrome associated with working hours stratified by age among men in Japan, 2009

	**<40 years**		**≥40 years**	
	**(*n* = 334)**		**(*n* = 569)**	
**Working hours**	**OR ^a^**	**(95% CI)**	**OR ^a^**	**(95% CI)**
≥7 to 8 hours/day	1.00	Reference	1.00	Reference
>8 to 9 hours/day	0.62	(0.13–2.96)	2.02	(1.04–3.90)
>9 to 10 hours/day	1.93	(0.59–6.34)	1.21	(0.53–2.77)
>10 hours/day	0.77	(0.14–4.14)	3.14	(1.24–7.95)

*CI* confidence interval, *OR* odds ratio.

^a^ Adjusted for age (continuous), occupation, shift work, smoking status, frequency of alcohol consumption, and cohabiting status.

Finally, we restricted the analysis to technicians or skilled workers. It should be noted that these two categories comprised the majority of the subjects. We observed heterogeneous results across the two types of occupation, although the precision of the estimates was considerably low. Among technicians, the multivariate ORs for 8–9, 9–10, and >10 working hours/day were 2.00 (95% CI, 0.38–10.57), 1.36 (95% CI, 0.27–6.78), and 1.86 (95% CI, 0.39–8.86), respectively. By contrast, among skilled workers, the multivariate ORs for 8–9 and 9–10 working hours/day were 1.66 (95% CI, 0.80–3.45) and 1.45 (95% CI, 0.56–3.71), respectively (the OR for >10 h/day was not available owing to a lack of data).

### Characteristics of unanalyzed participants

The mean age among the excluded 381 men was 51.5 years, which was significantly older than the analyzed participants. The means of working hours and sleeping hours were 9.0 and 6.5, respectively: the former was significantly shorter, and the latter significantly longer, than the analyzed participants. Information relating to metabolic syndrome was unavailable for 360 subjects; among the remaining 21 men, only one met the criteria for metabolic syndrome. Although there was no significant difference in occupation, there was a significant difference in cohabiting status: 344 (91.0%) unanalyzed subjects cohabited whereas 731 (78.4%) analyzed subjects cohabited.

## Discussion

To the best of our knowledge, this is the first study to investigate the association between long working hours and metabolic syndrome. This study suggests that compared with subjects who work 7–8 h/day, those reporting long working hours (>10 h/day) had over double the odds of metabolic syndrome after adjusting for age, occupation, shift work, smoking status, frequency of alcohol consumption, and cohabiting status. Similar patterns were observed among non-shift workers. In the stratified analysis by age-group, long working hours were associated with a higher risk of metabolic syndrome among older subjects, although no clear association was observed among younger subjects.

It should be noted that we observed no substantial increase in risk of metabolic syndrome among subjects who worked ≤10 h/day, although we observed a two-fold increase in odds among those who worked >10 h/day. Thus, the present findings suggest 10 h/day as a possible trigger level of working hours for metabolic syndrome among Japanese male workers. In line with the present findings, Kawakami et al. [13] showed in their prospective cohort study of Japanese men that those who worked ≥50 h/month (≥10 h/day) had a 3.7 times higher risk of developing non-insulin-dependent diabetes than those who did not undertake overtime work. A six-year study of women in the Nurses’ Health Study II indicated that subjects who worked ≥41 h/week were associated with a higher risk of type 2 diabetes than those who worked 21–40 h/week [14]. In contrast, Nakanishi et al. [15] reported that among Japanese male workers, the relative risk of type 2 diabetes significantly decreased among those who worked >10 h/day compared with those who worked 7–8 h/day. Another case–control study in Japanese men has also shown that working >11 h/day is associated with more than double the risk of myocardial infarction compared with men whose mean working time was 7–9 h/day [16]. By applying the benchmark dose approach, Suwazono et al. [31] examined the benchmark durations of working hours for development of fatigue symptoms in Japanese workers. Assuming a condition of worst job stress, they demonstrated that the threshold number of working hours among men was approximately 10 h/day with a benchmark response of 5%. On the other hand, it can be argued from our findings that 8 h/day could also be a possible trigger level. Indeed, the point estimates of ORs in Models 1 and 2 were the highest among those who worked 8–9 h/day, and we observed a dip among those who worked 9–10 h/day in Models 1 to 3. The increased ORs among those who worked 8–9 h/day, however, could be partially explained by the effect of the Labour Standards Law, which stipulates that working hours be limited to 40 h/week. Workers who developed health problems may have been required to restrict their working hours to avoid further adverse health outcomes arising from long working hours. This so-called healthy worker effect could also have been induced by self-regulation among subjects who have higher control over their work schedule, and this tendency may be more pronounced in workplaces with a higher social network or social capital [32-34]. Although the evidence remains inconclusive, our findings appear to suggest that a 10-hour working day is the trigger level for a significant increase in the risk of metabolic syndrome among Japanese male workers aged ≥40 years.

One plausible explanation for the link between long working hours and metabolic syndrome may be insufficient recovery time, primarily caused by shortened sleeping hours as a result of the long working hours [20]. Indeed, long working hours (≥40 h/week) have been shown to be a risk factor for the development of shortened sleeping hours (<6–7 h/day) [35-37], which may increase the risk of obesity [38-40]. In addition, since short sleep duration has been found to be a risk factor for diabetes, hypertension, and all-cause mortality [41], long working hours could increase the risk of chronic medical conditions by shortening sleep duration [42]. A case–control study in Japan indicated that long working hours and sleep deprivation are associated with an increased risk of acute myocardial infarction [43]. When we additionally adjusted for sleeping hours as a supplementary analysis, we observed a slight attenuation in the positive association between working hours and metabolic syndrome. Although careful consideration is required with its interpretation, this finding may support the above-mentioned hypothesis [44,45].

Another plausible explanation for the present findings may be that long working hours could modify lifestyles, resulting in a lack of time or energy available for healthy activities. For example, a recent review showed that leisure-time physical activity was less common among those working more than 45–50 h/week [46]. Indeed, lifestyle factors including physical activities are inter-correlated, and there may be complex interactions among them. A recent study in Japan reported that adherence to healthy lifestyles (such as regular physical activity and healthy eating) was associated with a lower risk of metabolic syndrome [47]. Although information relating to such factors was not available in the present study, it is likely that lifestyle factors are influenced by the work environment, especially long working hours.

We observed heterogeneous effects of long working hours on metabolic syndrome across different age-groups, and a significant association was noted only among subjects aged 40 years or older. This finding may well reflect the long-term effects of long working hours among older subjects. Indeed, it is less likely that long working hours could increase the risk for metabolic syndrome in the short term. However, we should note that the present findings do not necessarily imply that long working hours among younger workers are permissible: even shortly after joining a company, long working hours may potentially influence the future risk of metabolic syndrome by inducing behavioral and lifestyle changes. We should also note that the prevalence of metabolic syndrome among subjects aged <40 years was lower than that among the subjects aged ≥40 years or older (6.8% and 14.8%, respectively), which is consistent with the findings of the National Health and Nutrition Survey in Japan [4]. Among younger subjects, it may be that factors in operation before they start work at a company (e.g., family background and childhood lifestyle) determine the risk for metabolic syndrome. From a life-course perspective, future studies are warranted to investigate both pre- and post-employment risk factors (including working hours) for metabolic syndrome.

On a related issue, previous studies have reported that shift workers are at higher risk of developing hypertension and obesity [48,49]. In addition, other cohort studies have reported an association between shift work and abnormal glucose metabolism [50,51], which may suggest an association between shift work and metabolic syndrome. When we restricted the present analysis to non-shift workers, we observed similar patterns to those in the total sample. However, partly because of the small sample size, we were unable to assess the relationship among shift workers. Additional studies are needed to examine how specific aspects of work schedules, including the length and frequency of rotation, influence the risk of metabolic syndrome.

Interestingly, when we further examined the association between working hours and metabolic syndrome by restricting the analysis to technicians and skilled workers, we observed heterogeneous results with respect to occupation. Given the considerably low precision of the estimates, it is necessary to exercise caution when interpreting the results of this supplementary analysis. This finding may, however, indicate the significance of occupation when examining the association between working hours and metabolic syndrome. This point has been less carefully examined and, given the widening health disparities across occupations for both sexes during the recent economic downturns in Japan [52,53], future studies are necessary to investigate this possible heterogeneous effect.

### Study limitations

First, working hours were measured only at a single time point, and we could not assess the history of working hours among the subjects. It is likely that working hours vary substantially depending on economic conditions, and further studies with repeated measurement of working hours may yield important findings in this regard. Second, since working hours were assessed by self-reported questionnaire, the possibility of misclassification cannot be ruled out. However, the misclassification of working hours would be non-differential to metabolic syndrome, which may have attenuated the present findings. Third, owing to lack of information about the use of medication, the possibility of misclassification of diseased as non-diseased conditions cannot be ruled out. This misclassification, however, would be non-differential, which may have attenuated the present results. Fourth, because of the cross-sectional study design, we cannot exclude the possibility of reverse causation. However, it is implausible that the presence of metabolic syndrome caused the working hours of subjects to increase. Fifth, information about physical activities and job stress was not available. Since these factors potentially mediate the association between working hours and metabolic syndrome, their assessment would promote our understanding of this cause-effect relationship [54-56]. Sixth, half of the study subjects were skilled workers (49.7%), which, despite the high response rate (96.2%), may reduce the generalizability of our findings to Japanese male workers as a whole.

## Conclusions

We have found that compared with normal working hours (7–8 h/day), long working hours (>10 h/day) are associated with more than double the odds of metabolic syndrome among Japanese male workers. Though significant efforts are needed to manage the individual abnormalities of metabolic syndrome and the prevention of metabolic syndrome is essential in reducing cardiovascular disease [2], the findings of the present study may indicate the necessity of a well-designed population strategy for metabolic syndrome [57], given the existing culture of long working hours in Japan. Despite the working-hour regulations specified by the current Labour Standards Law, there is an exceptional rule whereby an employer can extend the working hours by an additional 45 h/month. Further studies are required to examine whether proper management of working hours can serve as a primary preventive measure for metabolic syndrome.

## Abbreviations

CI: Confidence interval; HDL: High-density lipoprotein; OR: Odds ratio.

## Competing interests

The authors declare that they have no competing interests.

## Authors’ contributions

ST and ES conceived the study and participated in its design. TK and ES performed the statistical analysis, interpreted the results, and drafted the manuscript. ST and HD contributed to interpretation of the results and critically revised the manuscript. All authors read and approved the final manuscript.

## Pre-publication history

The pre-publication history for this paper can be accessed here:

http://www.biomedcentral.com/1471-2458/12/395/prepub

## Supplementary Material

Additional file 1 **Table S1.** Odds ratios for metabolic syndrome associated with working hours among non-shift male workers, Japan, 2009. As a supplementary analysis, we restricted the subjects to non-shift workers to examine whether the associations between long working hours and metabolic syndrome were modified by shift work. **Table S2**. Odds ratios for metabolic syndrome associated with working hours stratified by age (<45 years vs. ≥45 years) among men in Japan, 2009. As a supplementary analysis, we stratified the subjects by age (<45 years vs. ≥45 years). **Table S3**. Odds ratios for metabolic syndrome associated with working hours stratified by age (<50 years vs. ≥50 years) among men in Japan, 2009. Description of data: As a supplementary analysis, we stratified the subjects by age (<50 years vs. ≥50 years). Click here for file
