# Supplementary material for: Long working hours and metabolic syndrome among Japanese men: a cross-sectional study
Source: BMC Public Health. 2012 May 31;12:395. doi: 10.1186/1471-2458-12-395 (PMC3419617; doi:10.1186/1471-2458-12-395)
Supplement: Additional file 1 — Table S1. Odds ratios for metabolic syndrome associated with working hours among non-shift male workers, Japan, 2009. As a supplementary analysis, we restricted the subjects to non-shift workers to examine whether the associations between long working hours and metabolic syndrome were modified by shift work. Table S2. Odds ratios for metabolic syndrome associated with working hours stratified by age (<45 years vs. ≥45 years) among men in Japan, 2009. As a supplementary analysis, we stratified the subjects by age (<45 years vs. ≥45 years). Table S3. Odds ratios for metabolic syndrome associated with working hours stratified by age (<50 years vs. ≥50 years) among men in Japan, 2009. Description of data: As a supplementary analysis, we stratified the subjects by age (<50 years vs. ≥50 years). [file 1471-2458-12-395-S1.docx]

**Additional files**

| **Table S1**  Odds ratios for metabolic syndrome associated with working hours among non-shift male workers, Japan, 2009 | | | | | | | | |
| --- | --- | --- | --- | --- | --- | --- | --- | --- |
|  | Model 1 ^a^  (*n*=688) | |  | Model 2 ^b^  (*n*=688) | |  | Model 3 ^c^  (*n*=670) | |
| Working hours | OR | (95% CI) |  | OR | (95% CI) |  | OR | (95% CI) |
| ≥7 to 8 hours/day | 1.00 | Reference |  | 1.00 | Reference |  | 1.00 | Reference |
| >8 to 9 hours/day | 1.74 | (0.91-3.34) |  | 1.73 | (0.90-3.33) |  | 1.86 | (0.83-4.17) |
| >9 to 10 hours/day | 1.13 | (0.57-2.24) |  | 1.26 | (0.63-2.53) |  | 1.46 | (0.57-3.74) |
| >10 hours/day | 1.36 | (0.71-2.58) |  | 1.65 | (0.85-3.20) |  | 2.28 | (0.88-5.90) |
| CI, confidence interval; OR, odds ratio. | | | | |  |  |  |  |
| ^a^ Crude model.  ^b^ Adjusted for age (continuous).  ^c^ Adjusted for age (continuous), occupation, smoking status, frequency of alcohol consumption, and cohabiting status. | | | | | | | | |
|  |  |  |  |  |  |  |  |  |

| **Table S2**  Odds ratios for metabolic syndrome associated with working hours stratified by age (<45 years vs. ≥45 years) among men in Japan, 2009. | | | | | |
| --- | --- | --- | --- | --- | --- |
|  | <45 years  (*n*=495) | |  | ≥45 years  (*n*=408) | |
| Working hours | OR ^a^ | (95% CI) |  | OR ^a^ | (95% CI) |
| ≥7 to 8 hours/day | 1.00 | Reference |  | 1.00 | Reference |
| >8 to 9 hours/day | 0.79 | (0.29-2.17) |  | 2.82 | (1.31-6.05) |
| >9 to 10 hours/day | 0.79 | (0.29-2.17) |  | 2.40 | (0.96-6.03) |
| >10 hours/day | 0.81 | (0.26-2.52) |  | 5.13 | (1.65-15.99) |
| CI, confidence interval; OR, odds ratio. | | | | | |
| ^a^ Adjusted for age (continuous), occupation, shift work, smoking status, frequency of alcohol consumption, and cohabiting status. | | | | | |
|  |  |  |  |  |  |

| **Table S3**  Odds ratios for metabolic syndrome associated with working hours stratified by age (<50 years vs. ≥50 years) among men in Japan, 2009. | | | | | |
| --- | --- | --- | --- | --- | --- |
|  | <50 years  (*n*=582) | |  | ≥50 years  (*n*=322) | |
| Working hours | OR ^a^ | (95% CI) |  | OR ^a^ | (95% CI) |
| ≥7 to 8 hours/day | 1.00 | Reference |  | 1.00 | Reference |
| >8 to 9 hours/day | 0.68 | (0.26-1.78) |  | 3.33 | (1.51-7.38) |
| >9 to 10 hours/day | 0.85 | (0.34-2.13) |  | 1.97 | (0.70-5.49) |
| >10 hours/day | 0.94 | (0.34-2.61) |  | 4.86 | (1.20-19.64) |
| CI, confidence interval; OR, odds ratio. | | | | | |
| ^a^ Adjusted for age (continuous), occupation, shift work, smoking status, frequency of alcohol consumption, and cohabiting status. | | | | | |
|  |  |  |  |  |  |
